# A Hückel Model for the Excited-State Dynamics of a Protein Chromophore Developed Using Photoelectron Imaging

**DOI:** 10.1021/acs.accounts.1c00780

**Published:** 2022-02-17

**Authors:** Cate S. Anstöter, Jan R. R. Verlet

**Affiliations:** Department of Chemistry, Durham University, Durham DH1 3LE, United Kingdom

## Abstract

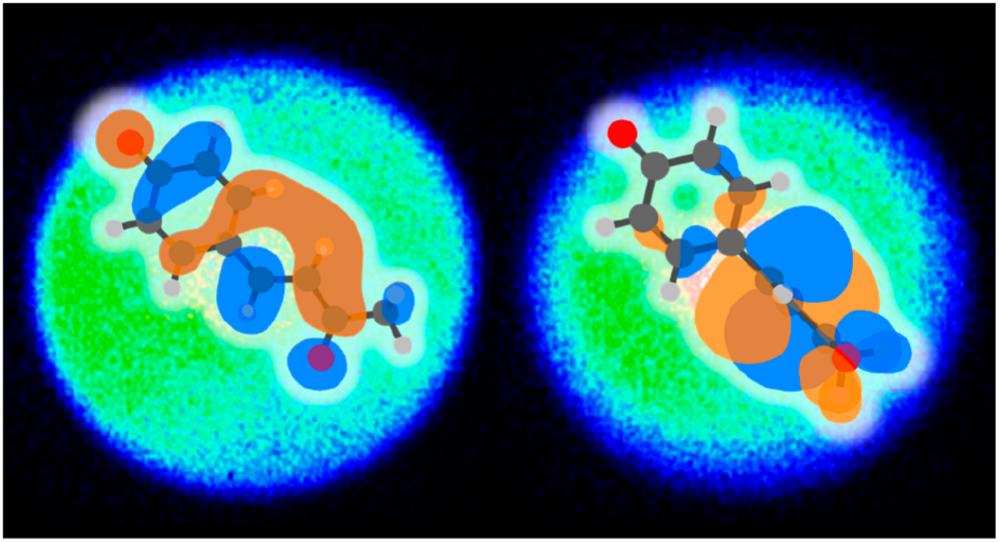

Chemistry can be described as the movement of nuclei within molecules and the concomitant instantaneous change in electronic structure. This idea underpins the central chemical concepts of potential energy surfaces and reaction coordinates. To experimentally capture such chemical change therefore requires methods that can probe both the nuclear *and* electronic structure simultaneously and on the time scale of atomic motion. In this Account, we show how time-resolved photoelectron imaging can do exactly this and how it can be used to build a detailed and intuitive understanding of the electronic structure and excited-state dynamics of chromophores. The chromophore of the photoactive yellow protein (PYP) is used as a case study. This chromophore contains a para-substituted phenolate anion, where the substituent, R, can be viewed as an acrolein derivative. It is shown that the measured photoelectron angular distribution can be directly related to the electronic structure of the para-substituted phenolate anion. By incrementally considering differing R groups, it is also shown that these photoelectron angular distributions are exquisitely sensitive to the conformational flexibility of R and that when R contains a π-system the excited states of the chromophore can be viewed as a linear combination of the π* molecular orbitals on the phenolate (π_Ph_*) and the R substituent (π_R_*). Such Hückel treatment shows that the S_1_ state of the PYP chromophore has predominantly π_R_* character and that it is essentially the same as the chromophore of the green fluorescent protein (GFP). The S_1_ excited-state dynamics of the PYP chromophore probed by time-resolved photoelectron imaging clearly reveals both structural (nuclear) dynamics through the energy spectrum and electronic dynamics through the photoelectron angular distributions. Both motions can be accurately assigned using quantum chemical calculations, and these are consistent with the intuitive Hückel treatment presented. The photoactive protein chromophores considered here are examples of where a chemists’ intuitive Hückel view for ground-state chemistry appears to be transferable to the prediction of photochemical excited-state reactivity. While elegant and insightful, such models have limitations, including nonadiabatic dynamics, which is present in a related PYP chromophore, where a fraction of the S_1_ state population forms a nonvalence (dipole-bound) state of the anion.

## Key References

- AnstöterC. S.; CurchodB. F. E.; VerletJ. R. R.Geometric and Electronic Structure Probed along the Isomerisation Coordinate of a Photoactive Yellow Protein Chromophore. Nat. Commun.2020, 11( (1), ), 282710.1038/s41467-020-16667-x32499507PMC7272410.^1^*The electronic and nuclear dynamics are resolved along the isomerization coordinate of the PYP chromophore using time-resolved photoelectron imaging and electronic structure calculations*.
- AnstöterC. S.; DeanC. R.; VerletJ. R. R.Chromophores of Chromophores: A Bottom-up Hückel Picture of the Excited States of Photoactive Proteins. Phys. Chem. Chem. Phys.2017, 19( (44), ), 29772–2977910.1039/C7CP05766K28937696.^2^*A Hückel picture to explain the excited states of the PYP and GFP chromophores is presented on the basis of a linear combination of the lowest unoccupied molecular orbitals on phenolate and the para-substituted fragment*.
- AnstöterC. S.; DeanC. R.; VerletJ. R. R.Sensitivity of Photoelectron Angular Distributions to Molecular Conformations of Anions. J. Phys. Chem. Lett.2017, 8( (10), ), 2268–227310.1021/acs.jpclett.7b0072628471670.^3^*This study shows how the photoelectron emission angle for a para-substituted phenolate anion depends sensitively on the conformation of the substituent*.
- BullJ. N.; AnstöterC. S.; VerletJ. R. R.Ultrafast Valence to Non-Valence Excited State Dynamics in a Common Anionic Chromophore. Nat. Commun.2019, 10( (1), ), 582010.1038/s41467-019-13819-631862884PMC6925192.^4^*The isomerization dynamics in a PYP chromophore is shown to compete with internal conversion to a nonvalence dipole-bound state of the anion, which can be clearly identified through time-resolved photoelectron imaging*.

## Introduction

Light-driven processes are common in biology and are central to phototaxis, vision, and photosynthesis. While the biological response involves large proteins and protein complexes, the initial photoinduced process typically involves a small molecular chromophore that acts as a light-sensitive transducer to mechanically initiate a large-scale response.^5^ This transduction often takes the form of isomerization about specific bonds.^6^ Understanding the initial motion and how light activates biological function has been widely studied for many years and was among the first processes to be studied in real time.^7^ An ultimate goal is to completely understand the excited-state dynamics taking place in the chromophore and its immediate surroundings so that the chromophore may be adapted and controlled to drive a specific outcome, be it more efficient transduction to mechanical motion (e.g., for vision) or enhanced fluorescence (e.g., for signaling or communication). To gain such insight, studying the chromophore in isolation from the protein environment has been very useful.^8^ Using this bottom-up approach offers a window into the intrinsic photophysics of the chromophore and is amenable to high-level calculations, the combination of which can yield the foundational understanding of the initial chemical dynamics. Over the years, virtually all studies with these goals in mind have focused on how the nuclei move following photoexcitation. However, ignoring the electronic evolution essentially ignores the basic premise of chemistry—that the electronic character adiabatically evolves with nuclear motion (the Born–Oppenheimer approximation). To fully understand chemical dynamics, both the electronic and nuclear dynamics should be probed in unison. There have been exquisite experiments in recent years to achieve this goal, but most have focused on very small molecular species through complex experimental methods that are not easily extended to the size of the chromophores involved in photobiology.^9−13^ We have recently developed methods to bridge this by using time-resolved photoelectron imaging of anions in conjunction with computational methods. In this Account, we describe how such experiments provide the required information and how this has offered an understanding of the structure of biochromophores based on substituted phenolate anions.

**Figure 1:**
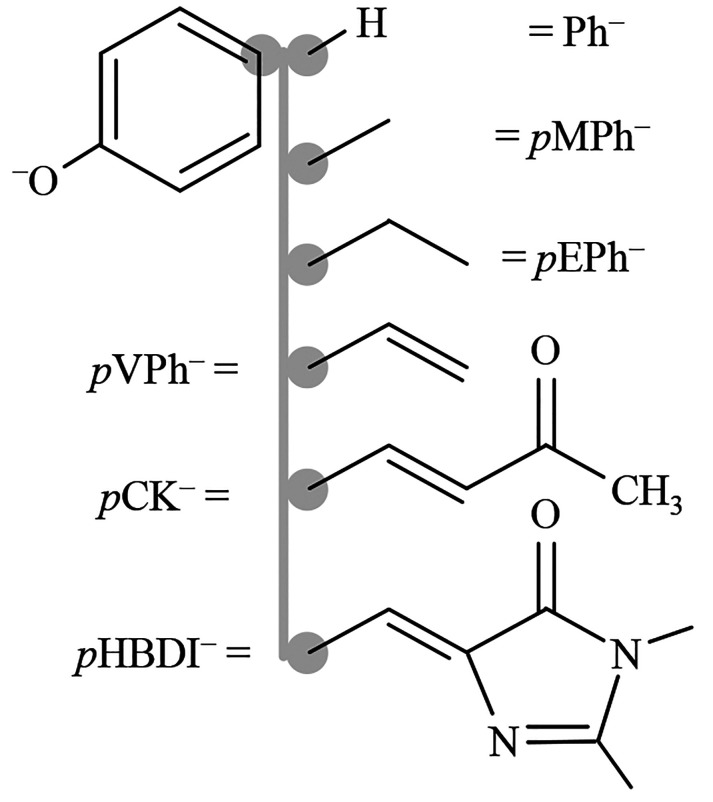
Structures of para-substituted (with R groups) phenolate anions considered: Ph^–^ = phenolate, *p*MPh^–^ = *p*-methylphenolate, *p*EPh^–^ = *p*-ethylphenolate, *p*VPh^–^ = *p*-vinylphenolate, *p*CK^–^ = *p*-coumaric ketone, and *p*HBDI^–^ = *p*-hydroxybenzylidene-1,2-dimethylimidazoline.

The phenolate anion is a common motif in nature and photoactive proteins, with well-known examples including the green fluorescent protein (GFP) and the photoactive yellow protein (PYP). Here, we will focus on the latter. PYP is a protein that acts to induce negative phototaxis in response to blue light in several bacterial organisms.^14^ The chromophore of PYP is a *p*-coumaric acid that is commonly modeled by a *p*-coumaric ketone (*p*CK^–^, Figure 1).^15^ PYP and its chromophore have often been used to demonstrate new experimental methods to probe its nuclear dynamics, with examples including time-resolved photoelectron spectroscopy,^16^ fifth-order time-domain Raman spectroscopy,^17^ and serial time-resolved X-ray diffraction at free-electron lasers.^18^ Here, we develop the former of these further and show how additional differential measurements on the photoelectrons offer concomitant insight into the electronic evolution. In addition, we unpick the electronic structure through the use of a simple model based on Hückel theory in which the chromophore is built up of molecular subunits. This model offers useful and intuitive chemical insight, especially with a view to designing and modifying the photophysical properties of chromophores.

## Photoelectron Imaging of Anions Formed by Electrospray Ionization

Photoelectron spectroscopy of gas-phase species determines the binding energy of electrons in molecules. Within a Koopmans’ picture, it effectively measures the orbital energy difference between a molecule with *N* and a molecule with *N* – 1 electrons. With the advent of charged-particle imaging and specifically velocity-map imaging,^19^ the already differential method of photoelectron spectroscopy gained a further dimension as both the magnitude and direction of photoelectron velocity vectors could be measured. The photoelectron angular distribution (PAD) is sensitive to the molecular orbital from which the electron is detached. In the molecular frame, this correlation is very well defined. However, even in a laboratory frame, despite the averaging that takes place over the initial orientational distributions, the PADs retain information about the electronic structure,^20^ and this information will be discussed here for chromophores based on para-substituted phenolate anions. The PADs are generally quantified using an anisotropy parameter, β_2_, which can vary from −1 to +2 for a single-photon transition: when β_2_ = +2, electrons are emitted predominantly parallel to the polarization axis of the light, **ε**; when β_2_ = −1, electrons are emitted predominantly perpendicular to **ε**; and when β_2_ = 0, emission is isotropic.^20,21^

There are some key benefits to probing anions (rather than more commonly studied neutral molecules). First, because the electron affinity of a molecule is generally much lower than its ionization energy, detaching an electron from an anion requires lower photon energies (typically in the range of <5 eV).^22,23^ Second, the fact that the anion is charged allows for mass selection prior to its photoelectron spectroscopy so that the sample is pure (although isomers/conformers and isobaric species could be present). Third, ion sources such as electrospray ionization and matrix-assisted laser desorption can be used to generate the anions, opening up a vast range of molecular systems that would otherwise not be possible to study using commonly used molecular beam methods for neutral molecules. Details of our home-built instrument that incorporates electrospray ionization with time-of-flight mass spectrometry and velocity map imaging have been described in detail elsewhere.^24,25^

## Photoelectron Angular Distributions as a Window into Geometric and Electronic Structure

**Figure 2:**
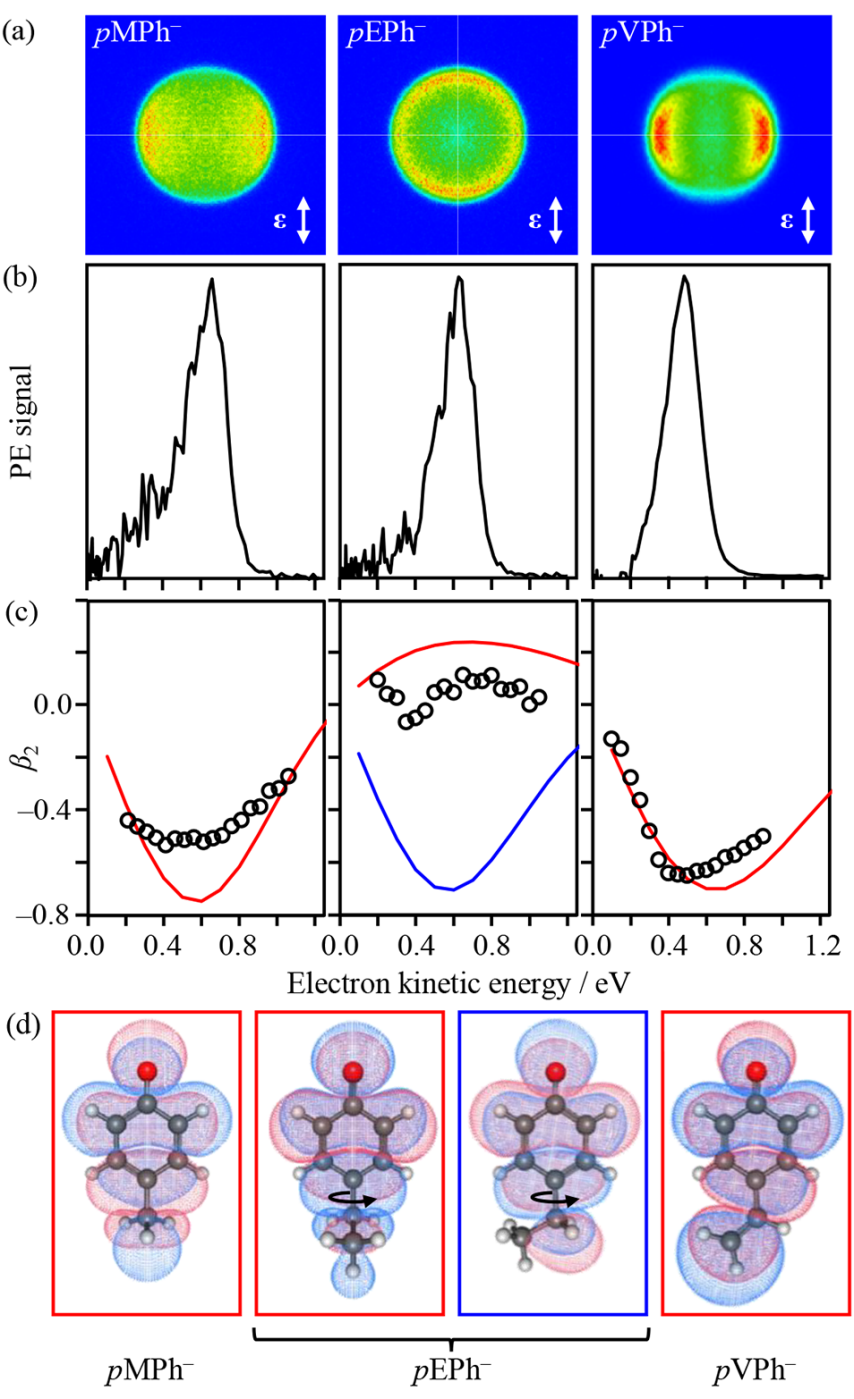
Photoelectron spectroscopy of para-substituted phenolates. (a) Photoelectron images of *p*-methylphenolate, *p*-ethylphenolate, and *p*-vinylphenolate taken at *hv* = 2.85 eV with the polarization axis shown by double arrows. (b) Corresponding photoelectron spectra. (c) β_2_ parameters from individual measurements (circles) at differing *hv* values and predicted (lines) by using the Dyson orbitals shown in (d). Adapted with permission from ref (3). Copyright 2017 American Chemical Society.

The PYP chromophore is a para-substituted phenolate (Figure 1), and we therefore start by considering the effect of substitution on the photoelectron spectra and PADs. The photoelectron images and spectra of the phenolate anion with a para-substituted methyl (*p*MPh^–^), ethyl (*p*EPh^–^), and vinyl (*p*VPh^–^) group are shown in Figure 2.^2^ The photoelectron images (Figure 2(a)) were taken at *hv* = 2.85 eV, and **ε** is indicated. The only available channel for detachment at this energy is to the ground state of the neutral molecule. In all three cases, the photoelectron images have similar radial components. Indeed, when extracting the photoelectron spectra from these images (Figure 2(b)), the overall spectral shape and binding energies of the photoelectron spectrum from the three molecular anions are very similar. However, the PADs associated with this seemingly similar detachment channel are very different across the three systems. For the detachment from *p*MPh^–^ and *p*VPh^–^, the overall emission is perpendicular to the polarization axis; β_2_ is negative. In contrast, the emission for *p*EPh^–^ appears to be significantly more isotropic, implying that β_2_ ≈ 0. In Figure 2(c), β_2_ is extracted from photoelectron images over a series of *hv* to allow the evolution of β_2_ as a function of the electron kinetic energy (eKE) to be extracted. Note that the eKE axes in Figure 2(b) and (c) are therefore not the same, with the latter corresponding to several measurements rather than the single measurement in the former. These plots clearly confirm the observation of differing behaviors (even qualitatively) of β_2_ for *p*EPh^–^ compared to those for *p*MPh^–^ and *p*VPh^–^. Data is considered only over the first 1 eV of the continuum; beyond this, metastable excited states of the anion (resonances) are accessed, leading to dramatic changes in the PADs.

Also shown in Figure 2(c) are the results of computed PADs, predicted using the Dyson orbital approach^26^ within the equation-of-motion ionization potential coupled-cluster singles and doubles formalism (EOM-IP-CCSD). The Dyson orbital, Ψ_D_, can be thought of as the one-electron orbital from which the electron is removed upon photodetachment. With a knowledge of Ψ_D_, the PADs can be calculated by using the eZDyson package developed by Krylov and co-workers.^27^ Details of the method and a benchmarking study exploring its application to anions^28^ have been described previously and will not be considered further here. Importantly, in the cases considered here, the PADs are always laboratory-frame observables. Note also that simple symmetry arguments can be formulated to account for the overall sign of β_2_, which can offer added insight without the need for high-level calculations.^29^

Figure 2(c) shows that the overall predicted trends for *p*MPh^–^ and *p*VPh^–^ are similar and in good overall agreement with the experimentally measured PADs. For *p*EPh^–^, the situation is more complex because this substituent has a torsional degree of freedom. Specifically, the ethyl group can be in the plane of the phenolate ring or perpendicular to this plane, with the latter being the lowest-energy conformation. The most striking observation here, however, is the sensitivity of the PADs to this conformational freedom.^3^ In Figure 2, the predicted β_2_ trend for the in-plane conformer of *p*EPh^–^ is similar to that of *p*MPh^–^ and *p*VPh^–^, whereas for the out-of-plane conformer, β_2_ is predicted to be slightly positive. The latter is in reasonable agreement with experiment and in accord with this conformer being the most prevalent in the anion distribution.^3^ The conformational differences are not apparent from the photoelectron spectra but are very clearly apparent from the PADs. Hence, the PADs are exquisitely sensitive to the small changes in electronic structure for these two conformers, with Ψ_D_ for all of the relevant species shown in Figure 2(d). In contrast, the photoelectron spectra are dictated by the Franck–Condon factors between the anion and neutral that are not sensitive to these conformation changes.

A natural extension of this work is now to track electronic evolution along a reaction coordinate in real time, which is described below.^1^ However, we first take a detour to consider the overall electronic structure of the PYP chromophore in the context of a simple Hückel theory picture.

## A Hückel Theory Model for Biochromophores

In general, biochromophores have extended polyenes (e.g., retinoids), aromatic rings (e.g., porphyrins), or both (e.g., GFP, PYP) as their chromophoric cores. In cases where both are present, one can view the electronic structure of the chromophore as a superposition of the molecular orbitals (MOs) associated with the ring and polyene. Such a view is complementary to high-level calculations, but with the benefit of its simplicity in terms of predicting how the electronic structure might change through minor chemical changes and therefore also its applicability to the broader chemical community.

An outstanding example of the success of simple Hückel approaches was demonstrated by Bravaya et al.,^30^ in which the electronic structure of the S_1_ state of differently colored photoactive proteins was rationalized through the use of a three-centered allyl radical in a simple Hückel framework and a particle in a box model. Utilizing a similar approach, Bochenkova et al. interpreted the electronic structure of the S_3_ state of the chromophore anion of GFP (*p*-hydroxybenzylidene-2,3-dimethylimidazolinone, *p*HBDI^–^) and its electronic excitation.^31^

**Figure 3:**
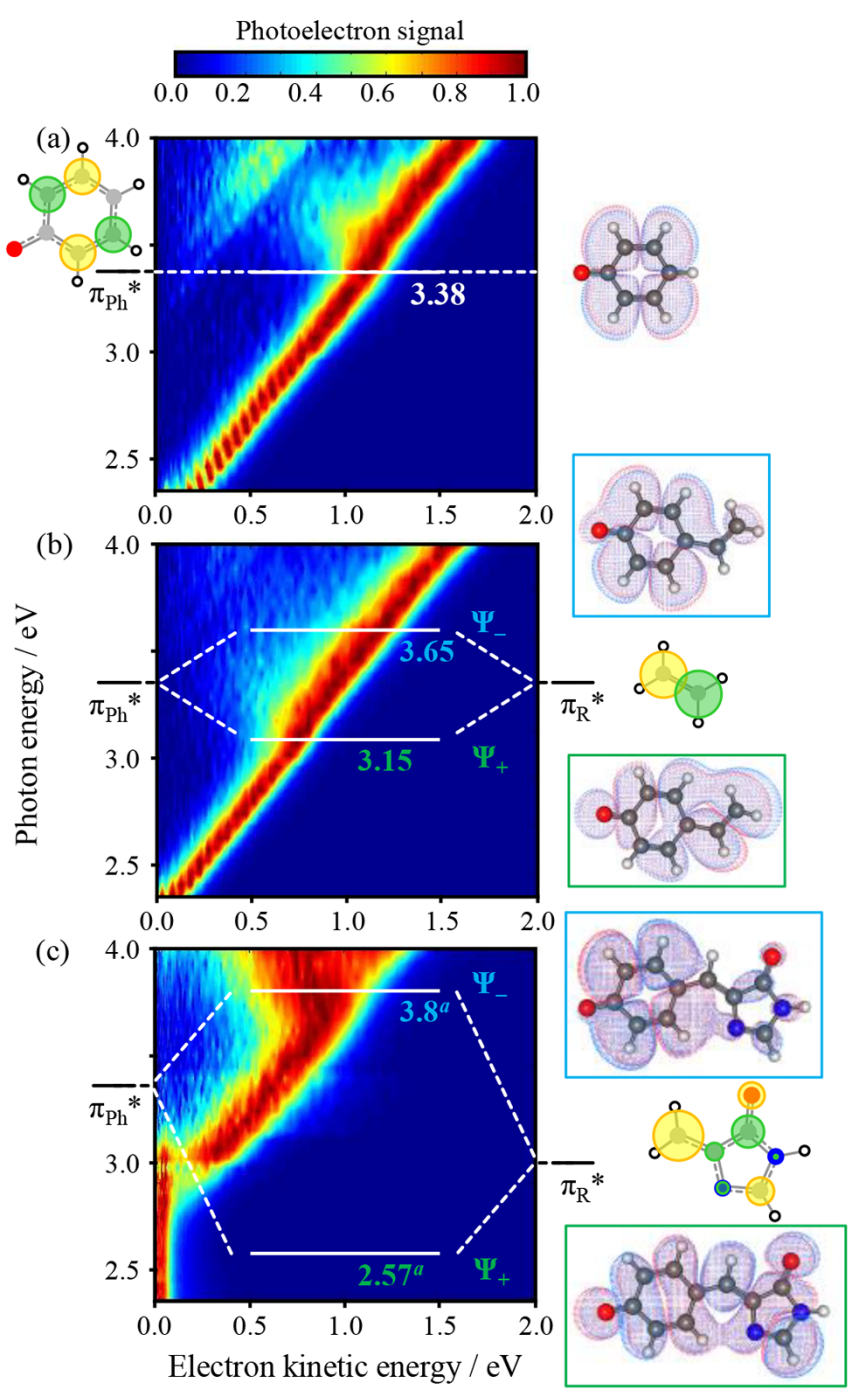
Two-dimensional photoelectron spectra of (a) phenolate, (b) *p*-vinylphenolate, and (c) *p*HDBI. Included is the energy of the resonances associated with the phenolate LUMO (π_Ph_*) and the LUMO energy of the para substituent R (π_R_*) along with the Hückel MOs. These combine as a linear combination of MOs to form the MOs Ψ_+_ and Ψ_–_ that are associated with the S_1_ and S_2_ excited states of the *p*-vinylphenolate and S_1_ and S_3_ of *p*HDBI. Adapted with permission from ref (2). Copyright 2017 Royal Society of Chemistry.

Taking inspiration, we applied a modified Hückel model to develop an understanding of the electronic structure of para-substituted phenolate anions. A comparison of the photoelectron spectra of the bare phenolate anion with *p*EPh^–^ and *p*VPh^–^, discussed in the previous section, and *p*CK^–^ (PYP) and *p*HBDI^–^ (GFP) provides us with bottom-up insight into the changing excited state as a function of substitution.^2^ The 2D photoelectron spectra for Ph^–^, *p*VPh^–^, and *p*HBDI^–^ are shown in Figure 3(a–c). All three are broadly similar: spectra are dominated by a feature for which the eKE increases linearly with increasing *hv*. This corresponds to the direct detachment channel discussed previously. The onset of a second direct detachment channel (in which the neutral is left in the first excited D_1_ state) is seen clearly for Ph^–^ starting at *hv* ≈ 3.2 eV. In addition to the direct detachment features, spectral broadening is seen for all anions over the *hv* range in which the signal from the high eKE channel shifts to lower eKE (e.g., at *hv* ≈ 3.7 eV in Figure 3(a)).^2^ Concurrent with the spectral broadening, abrupt changes were also observed in the PADs, indicative of a change in the molecular orbital (MO) from which the electron is lost. Both the broadened spectral signature and the abrupt changes in the PADs thus point to the presence of excited states of the anion, from which the electron is lost via autodetachment (i.e., resonances).^32−34^ The locations of these excited states are indicated in Figure 3.

Our interest is in understanding the electronic structure of the excited states. Consider the phenolate moiety. The lowest unoccupied MO corresponds to a π* orbital, π_Ph_*, and π_Ph_* ← π_Ph_ is the lowest-energy transition (i.e., S_1_ ← S_0_), as seen in Figure 3(a). Because π_Ph_* has a node along the O and the C in the para position, the addition of an alkyl group here does not affect π_Ph_* (σ–π separability), and the 2D photoelectron spectra and PADs of Ph^–^, *p*MPh^–^, and *p*EPh^–^ are all very similar. In the case of *p*VPh^–^ and *p*HBDI^–^, the R group has its own π system, with a corresponding empty antibonding MO, π_R_*. The electronic structure is now defined as a linear combination of π_Ph_* and π_R_*.

For *p*VPh^–^, two resonances are observed in Figure 3(b). First, consider the Hückel energy of π_Ph_*. Given that a node encompasses the O atom, the orbital energy of π_Ph_* is that of the π* MO of benzene, ε = α – β. When considering R, the C atom of the phenyl ring should be excluded. Thus, for *p*VPh^–^, R is ethene and π_R_* is the π* MO of ethene, which also has a Hückel energy, ε = α – β. Hence, the π_Ph_* and π_R_* MOs are degenerate in this simple picture, and a linear combination of π_Ph_* and π_R_* yields the two overall MOs: Ψ_±_ = 2^–1/2^π_Ph_* ± 2^–1/2^ π_R_*. This is in excellent agreement with the observations for *p*VPh^–^, which show that the two resonances are split by ∼0.2 eV on either side of the π_Ph_* resonance (i.e., β ≈ – 0.2 eV) (Figure 3(b)). It is also in agreement with electronic structure calculations,^2^ with the molecular orbitals associated with Ψ_+_ and Ψ_–_ shown in Figure 3(b).

The same Hückel framework can now be scaled to any R group to offer rather intuitive insight into the excited states of the chromophores. We first consider the GFP chromophore, *p*HBDI^–^, in Figure 3(c).^35^ In our model, R is 2-ethene-imidazole (methyl groups can be neglected because they have little effect on the π-electronic structure). The Hückel energy of π_R_* is ε = α – 0.35β. The resultant Ψ_+_ and Ψ_–_ MOs have a larger separation, and their character will be dictated by the coefficients in the linear combination. For Ψ_+_ (associated with S_1_), the π_R_* coefficients will be much larger than those for π_Ph_*. Hence, the S_1_ state will have predominantly π_R_* character. The higher-lying excited state associated with Ψ_–_ will have predominantly π_Ph_* character. These qualitative predictions are broadly consistent with both experiment and high-level electronic structure calculations. In *p*HBDI^–^, the S_1_ state is bound (i.e., it lies below the D_0_ level^36,37^) and indeed has predominantly π_R_* character according to high-level extended multiconfigurational quasi-degenerate perturbation theory (XMQCDPT2) calculations.^38^ The excited state associated with Ψ_–_ can also be seen in the experiment (Figure 3(c)) and by using computational chemistry, but it is the S_3_ state because there is an additional S_2_ core excited state that a simple Hückel model cannot account for.^31^ Here we focus on S_1_ but note that the higher-lying states are interesting in their own right from a photo-oxidation perspective in biomolecules.

**Figure 4:**
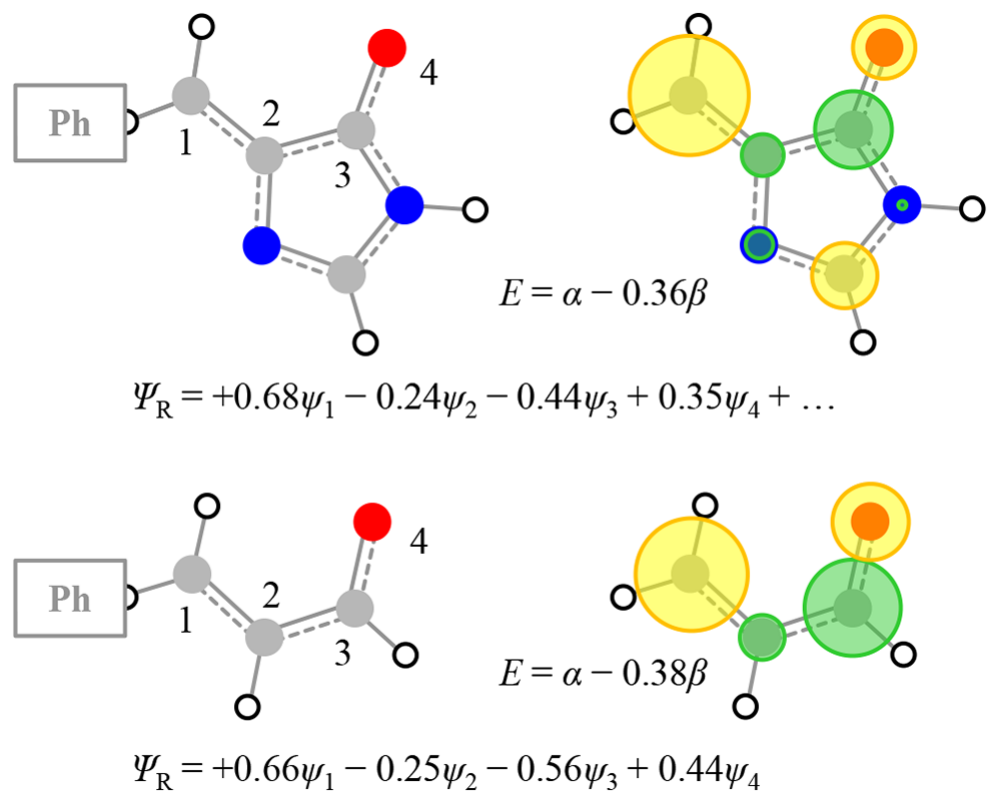
Structure and lowest unoccupied Hückel MOs of para substituents 2-ethene-imidazole and acrolein to represent the chromophores of GFP and PYP, respectively. Also shown are the orbital energy and the wave function of these MOs, showing the similarity between the two.

A remarkable conclusion from the Hückel picture is that the chromophores of GFP and PYP are essentially identical! For *p*CK^–^, R is acrolein (methyl vinyl ketone with the methyl group ignored), and in Figure 4, the π_R_* MOs of *p*HBDI^–^ and *p*CK^–^ are shown, demonstrating their striking similarity. The similarity comes about because of the very small coefficients on the N atoms in 2-ethene-imidazole, and this conclusion is in agreement with both the high-level calculations and experiment. The S_1_ state in *p*CK^–^ is similarly bound, and the S_3_ state can be identified with predominant π_Ph_* character.

Using the above insight and the conclusion that the S_1_ state of *p*CK^–^ has predominantly π_R_* character, we now return to considering how the electronic changes can be tracked along a reaction coordinate using *p*CK^–^ as an example.^1^

## Nuclear and Electronic Structure Simultaneously Measured along an Isomerization Coordinate

Although single-photon photoelectron imaging can probe the ground-state anion as described above, an excited state can be probed in a two-color pump–probe scheme. For *p*CK^–^, the S_1_ state dynamics were probed by initially exciting with a short pump pulse at 2.79 eV (444 nm) and subsequently probed at 1.55 eV (800 nm) by a second delayed short probe pulse. The excitation energy was finely tuned to excite the S_1_ state while minimizing direct detachment. The photoelectron image generated by the probe is a measure of the S_1_ state at the time it was probed, and its evolution can thus be tracked through the S_1_ + *hv*_probe_ → D_0_ + e^–^ detachment channel.^1^

**Figure 5:**
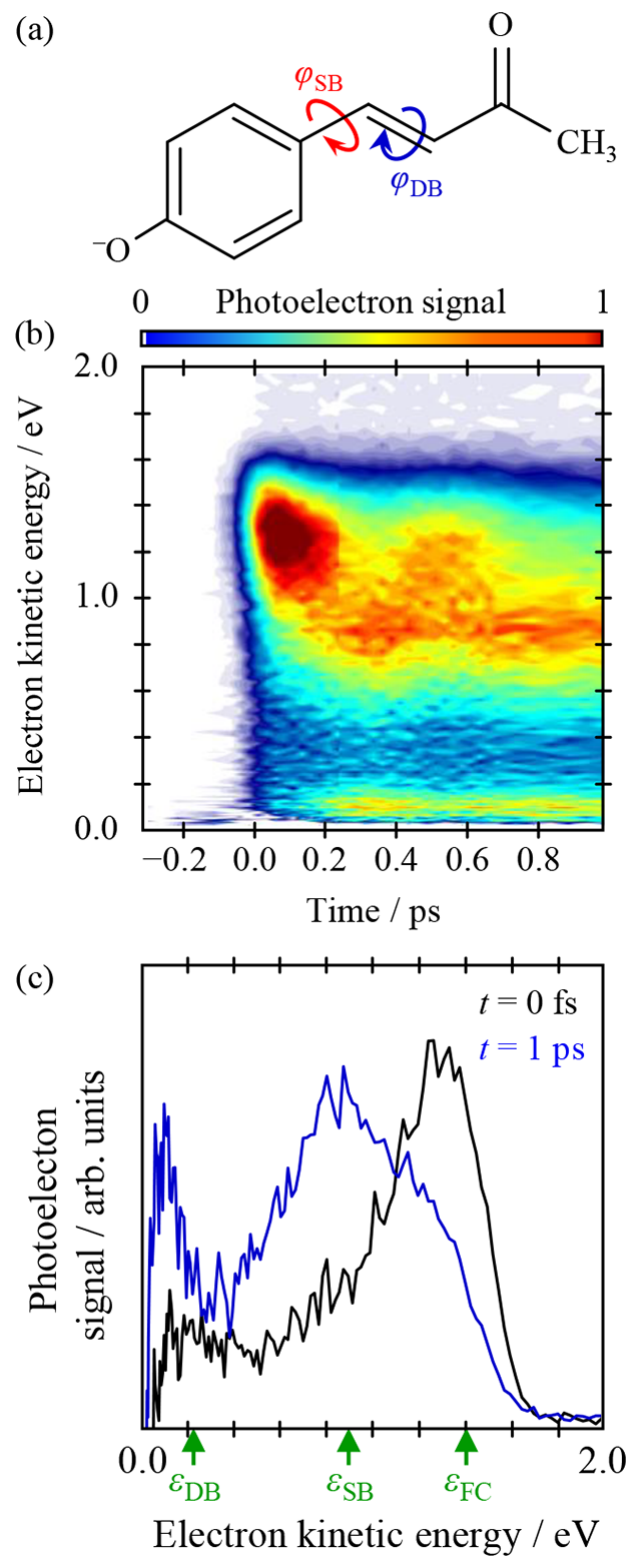
Time-resolved photoelectron spectroscopy of *p*CK^–^. (a) Structure of *p*CK^–^ and the bonds about which rotation can take place in the excited-state isomerization process. (b) Background-subtracted, time-resolved photoelectron spectra over the first picosecond following excitation at 2.79 eV and probing at 1.55 eV. (c) Individual photoelectron spectra at *t* = 0 (black) and 1 ps (blue), with green upward arrows indicating the expected maximum kinetic energy associated with detachment from the Franck–Condon geometry, ε_FC_, and the minimum following rotation about the single bond, ε_SB_, and about the double bond, ε_DB_. Adapted with permission from ref (1). Copyright 2020 the authors. Published by Nature Communications under a Creative Commons Attribution 4.0 International License http://creativecommons.org/licenses/by/4.0/.

Excitation to the S_1_ state leads to a weakening of the π system which enables isomerization. This isomerization can proceed along either the single bond connecting the phenyl ring to the para-substituted methyl vinyl ketone, φ_SB_, or along the double bond in the fragment, φ_DB_, as shown in Figure 5(a).^39^

Figure 5(b) shows the results of the experiment, where photoelectron spectra are presented as difference spectra in which any small signal at *t* < 0 has been subtracted from all time-resolved images to leave only the excited-state signals.^1^ At eKE < 0.05 eV, this leads to a negative signal, which arises from the bleaching of the small contribution of direct detachment and/or autodetachment. The main features of interest are associated with the positive photoelectron signal at eKE > 0.2 eV because these represent the evolution of the S_1_ state. At *t* = 0, the photoelectron signal peaks at eKE ≈ 1.3 eV. This then appears to decay and form a new feature peaking at eKE ≈ 0.8 eV but additionally shows coherence in which the population oscillates once between these two features, before settling. At times beyond 1 ps, the high eKE feature decays, leaving only the peak at eKE ≈ 0.8 eV, and this subsequently decays on a time scale of ∼120 ps.^1^ Here we focus on the first picosecond of the dynamics, and two representative photoelectron spectra at *t* = 0 and 1 ps are shown in Figure 5(c).

**Figure 6:**
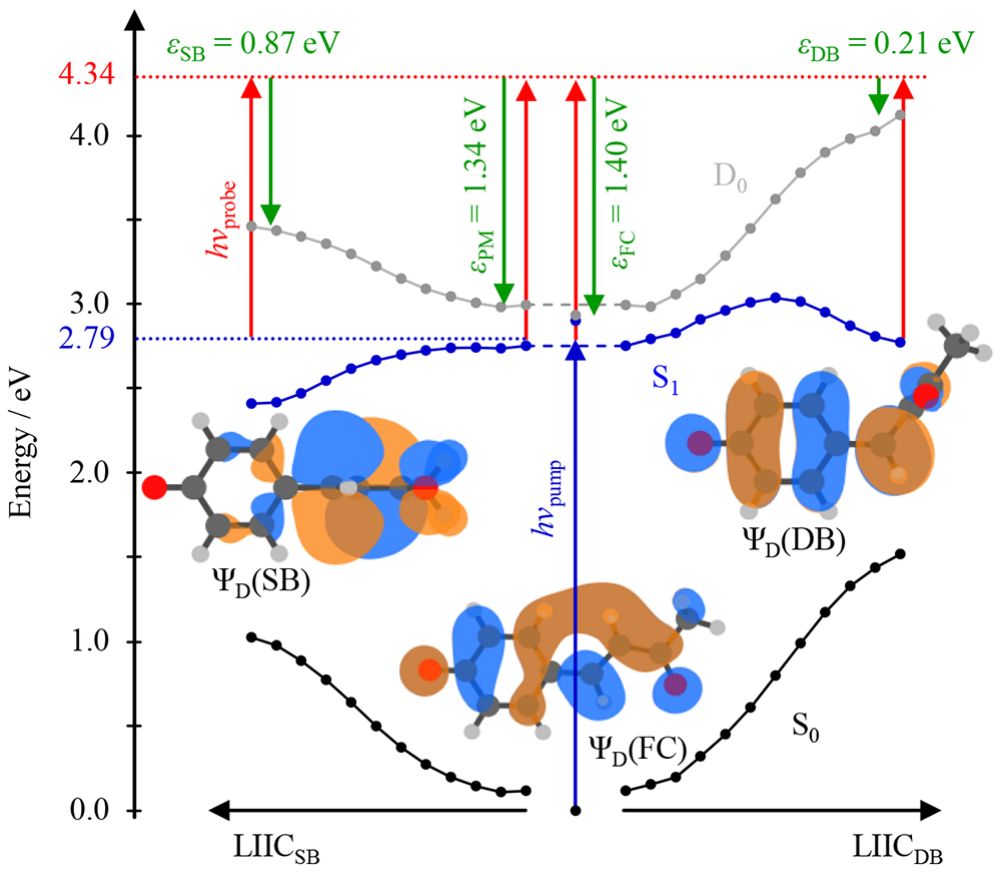
Potential energy curves for the S_0_ and S_1_ states of *p*CK^–^ and the D_0_ state of the neutral as a function of isomerization about the single bond (SB, left) and double bond (DB, right) using a linear interpolation of internal coordinates. Also shown are the Dyson orbitals at the key geometries: the minimum along SB rotation, Ψ_D_(SB), the Franck–Condon geometry, Ψ_D_(FC), and the minimum along DB rotation, Ψ_D_(DB). Vertical upward arrows indicate photoexcitation/detachment using the pump/probe pulse; downward arrows indicate the energy of the emitted electrons, with the maximal electron kinetic energies, ε, along the key geometries indicated. Adapted with permission from ref (1). Copyright 2020 the authors. Published by Nature Communications under a Creative Commons Attribution 4.0 International License http://creativecommons.org/licenses/by/4.0/.

To determine which isomerization coordinate (φ_SB_ or φ_DB_) is probed in the experiment, Figure 6 shows the potential energy curve along these two bond rotation coordinates. These were obtained by a linear interpolation in internal coordinates (LIIC) and a recalculation of the electronic energies of the S_1_ and D_0_ states at all points along the LIICs using multistate XMCQDPT2, with complete details given elsewhere.^1^ Spectroscopically, the photoelectron spectra are determined by the difference in energy between the S_1_ and D_0_ states, which evolves differently along the φ_SB_ and φ_DB_ coordinates. Specifically, at the Franck–Condon geometry, FC, we expect that probing the S_1_ state with 1.55 eV will lead to photoelectron signal with eKE extending to 1.40 eV. This is in agreement with the feature seen at *t* = 0 peaking at eKE ≈ 1.3 eV (Figure 5(c)). For the S_1_ state evolution about the φ_SB_ coordinate, leading to a twisted intermediate (SB), we anticipate that the photoelectron signal would extend to eKE = 0.87 eV, which is again in excellent agreement with the feature peaking at eKE ≈ 0.8 eV in Figure 5(c). In contrast, for S_1_ state evolution about the φ_DB_ coordinate, forming a twisted intermediate (DB), the signal should extend to eKE = 0.21 eV. Although there is an excited state signal observed at eKE < 0.2 eV, this arises as the probe from SB becomes resonant with the S_2_ state of *p*CK^–^ rather than from the detachment of DB, as shown by excited-state calculations.^1^

On this basis, we conclude that the photoexcited S_1_ state of *p*CK^–^ initially isomerizes about φ_SB_. This agrees with our calculations^1^ and others’^39,40^ calculations that find a barrier along the φ_DB_ coordinate. Our experiment shows that the nuclear wavepacket along this coordinate partially returns to the planar geometry once before dephasing.

**Figure 7:**
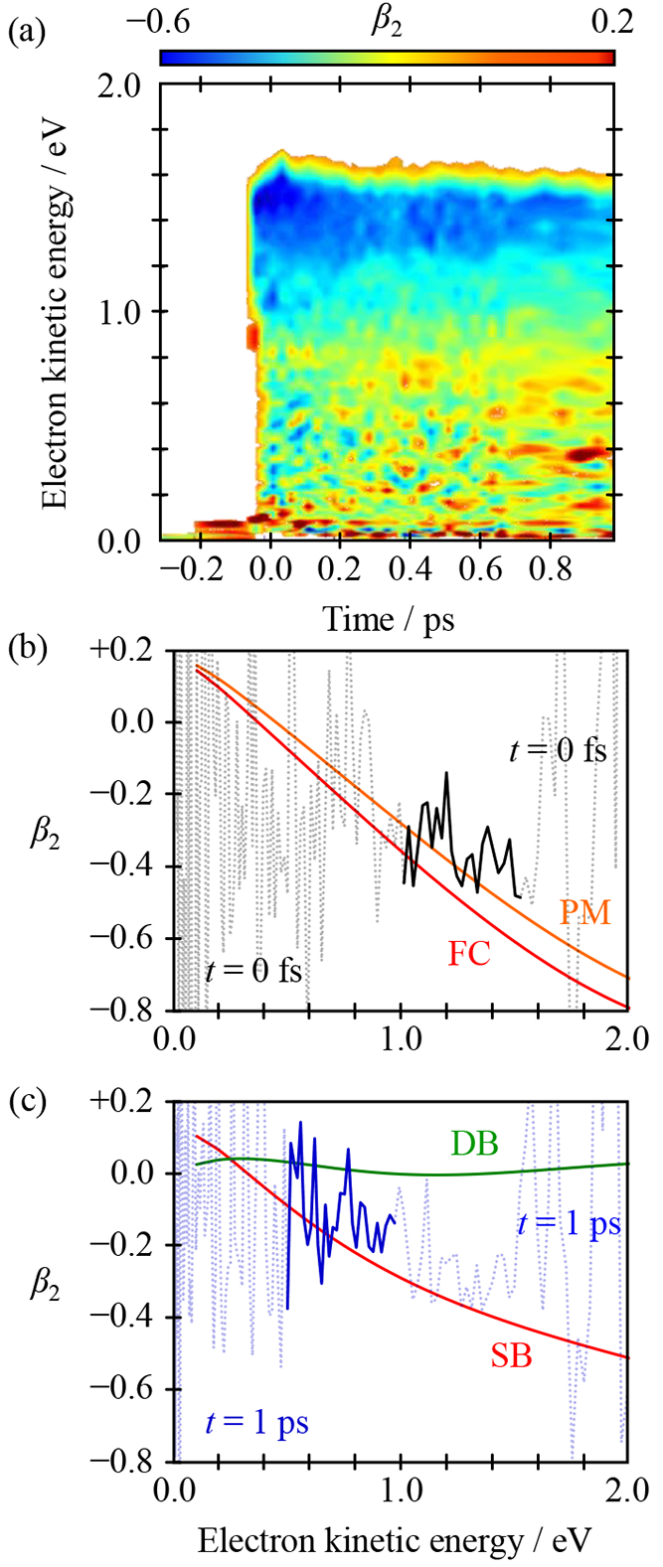
Time-resolved β_2_ spectra. (a) False color plot of β_2_ as a function of kinetic energy and time, which is directly comparable to the photoelectron signal in Figure 5(b). Slices of β_2_ spectra taken at (b) *t* = 0 ps (black solid line) and (c) *t* = 1 ps (blue solid line). Also shown on these is the predicted β_2_ spectrum from the Dyson orbitals for the key geometries in Figure 6. For *t* = 0 ps (b), this is comparable to the Franck–Condon geometry. For *t* = 1 ps (1), this is comparable to either SB rotation or DB rotation, with SB matching the data significantly better. Adapted with permission from ref (1). Copyright 2020 the authors. Published by Nature Communications under a Creative Commons Attribution 4.0 International License http://creativecommons.org/licenses/by/4.0/.

While the above arguments are solely based on energy arguments, our measurements also provide information about the electronic structure along the isomerization pathways. By analogy to the above case of *p*EPh^–^, rotation of the para-substituted fragment out of the plane of the ring may be expected to result in changes in the PADs.^3^Figure 7(a) shows the evolution of β_2_ as a function of both eKE and *t* (which relates directly to spectral changes in Figure 5(b)). In Figure 7(b) and (c), β_2_(eKE) is plotted at *t* = 0 and 1 ps, with the regions with high photoelectron signals shown as solid lines. We compare these to the predicted β_2_ from the relevant Dyson orbitals, which are shown in Figure 6.

At *t* = 0, we expect that the detachment will be from the S_1_ state in the FC geometry, so we use this Dyson orbital, Ψ_D_(FC), to calculate the β_2_(eKE), as shown in Figure 7(b). The overall agreement is very good and can be made even more convincing when we account for the initial motion on S_1_ that takes the FC geometry to a planar minimum on S_1_ (PM) prior to isomerization.^41^ As before, we consider both φ_SB_ and φ_DB_ coordinates leading to the SB and DB intermediates. The Ψ_D_ of these coordinates differ, so we might anticipate that their PADs will also differ. In Figure 7(c), we compare β_2_ computed from both Dyson orbitals with the experimental β_2_ at *t* = 1 ps. The β_2_ for SB is in much better agreement than that for DB, which is consistent with the conclusions from the photoelectron spectra. Hence, while the photoelectron spectra capture the nuclear evolution of the S_1_ state, the PADs clearly capture the electronic evolution. Taken holistically, we have therefore monitored the evolution of electronic character along a reaction coordinate, which is of course the basis of the Born–Oppenheimer approximation and the underpinning concept of a potential energy surface.

The predicted β_2_ in Figure 7 assumed a random spatial distribution of laboratory-frame molecules. In principle, photoexcitation to the S_1_ state could result in a prealigned distribution that is due to a defined transition dipole moment vector. Such alignment would be observable through higher-order anisotropy parameters (i.e., β_4_ in the present case), but these were found to be near zero, indicating that no substantial prealignment was present in these experiments, justifying the use of the one-photon β_2_ parameter.

We now briefly consider how our observations tie into the simple Hückel picture presented previously. We suggested that the S_1_ state of *p*CK^–^ could be viewed as a linear combination of π_Ph_* and π_R_*, where R represents methyl vinyl ketone (or acrolein). The Hückel energy of π_R_* is α – 0.38β (compared to α – β for π_Ph_*), so we expect that the S_1_ state *p*CK^–^ will have predominantly π_R_* character. Because the para bond is not directly involved in these considerations, it should come as no great surprise that there is little or no barrier to rotation about φ_SB_. Then, once rotation sets in, the π_Ph_* and π_R_* MOs become decoupled and the S_1_ electronic structure evolves to have almost exclusively π_R_* character in the SB geometry. Indeed, β_2_ predicted for the π_R_* fragment is in excellent agreement with that for the SB structure. Hence, the adiabatic evolution effectively involves a charge transfer from a delocalized MO of the FC geometry to a localized MO on the methyl vinyl ketone fragment. This evolution is primed by virtue of the π_R_* character of the FC S_1_ state as explained by the Hückel picture. This conclusion is pleasing because it suggests that simple Hückel theory arguments can be extended to excited states and their dynamics!

Of course, the Hückel approximations are drastic and have their limitations. One obvious limitation is nonadiabaticity, which is ultimately how the S_1_ state decays to the ground electronic state of *p*CK^–^. However, other nonadiabatic processes can take place, as we have observed in an experiment on the related chromophore in which the ketone is replaced by an ester, *p*CEs^–^.^4^ In this case, excitation to the analogous S_1_ state revealed a bifurcation of the wavepacket, with some population remaining on S_1_ and isomerizing (as seen in *p*CK^–^) and with another fraction nonadiabatically converting to a nonvalence dipole-bound state. While the time-resolved measurements capture these dynamics fully, evidence for the interplay of nonvalence states in the excited-state dynamics of anions can also be seen in the single-photon photoelectron spectra of many anions.^42−44^ Indeed, we have now seen the internal conversion from valence states to nonvalences states (and vice versa) in a range of molecular and cluster anionic system,^45−49^ where the excited state is close in energy to the detachment threshold. Why we see only the dipole-bound state in *p*CEs^–^ and not in *p*CK^–^ remains unknown. The interest in anion nonvalence states is growing because they are also implicated in electron capture processes and may be important even in condensed phases.^50^

## Conclusions and Outlook

The ability to probe both electronic and nuclear dynamics simultaneously has been one of the key goals of chemical dynamics. Here, we have done this on a relatively large biomolecule using a combination of established methods based on time-resolved photoelectron imaging as a detection method. The experimental method has much scope moving forward. Specifically, progressing to larger systems is straightforward using electrospray ionization. Although this will inevitably come with enhanced complexity of the results, gas-phase spectroscopy offers tools to attain exquisite control of the initial samples. For example, the temperature can be tuned from a few tens to hundreds of Kelvin using cryogenic ion traps,^51^ isomers can be preselected using ion mobility methods,^52^ specific modes can be pre-excited using light fields, surroundings can be introduced in a systematic and incremental manner, and the spectral/time resolution can be improved (at the expense of time/spectral resolution).^53^ On the theoretical front, the computation of the PADs remains difficult. At present, there is no consideration of the spread of configurations of the nuclei, although we have recently shown that the effects of internal motion due to temperature or dynamical effects can influence the computed PADs significantly.^28^ Finally, it is worth noting that there is still no robust way to compute the PADs for electron emission from resonances. Nevertheless, it is notable how well the electronic and nuclear dynamics can be tracked and correlated with computational results, offering much hope that these methods can also begin to offer new insights into more complex systems and nonadiabatic dynamics as well as the predominantly adiabatic dynamics discussed here.

We finish by marveling at the extent to which the underlying photodynamics of a biochromophore can be decomposed and understood using intuitive chemical models based on Hückel theory, which offers simple tools to be exploited by general chemists to develop the photoactive protein toolbox.
